# Photo-Enhanced Health Promotion Messages to Target Reduction in Dietary Sugar among Residents of Public Housing

**DOI:** 10.3390/nu15112601

**Published:** 2023-06-01

**Authors:** Mabeline Velez, Lisa M. Quintiliani, Yinette Fuertes, Annelli Román, Brenda Heaton

**Affiliations:** 1 Department of Health Policy & Health Services Research, Boston University Henry M. Goldman School of Dental Medicine, 560 Harrison Ave, 3rd Floor, Rm 336, Boston, MA 02118, USA; mabeline@bu.edu (M.V.); yfuertes@bu.edu (Y.F.); ajri@bu.edu (A.R.); 2 Department of Epidemiology, Boston University School of Public Health, Boston, MA 02118, USA; 3 Section of General Internal Medicine, Department of Medicine, Chobanian & Avedisian School of Medicine, Boston University and Boston Medical Center, Boston, MA 02215, USA; lisa.quintiliani@bmc.org

**Keywords:** text messages, health promotion/methods, internet interventions, digital health, health interventions, public housing

## Abstract

Interventions intended to reduce the consumption of dietary sugars among those population groups demonstrating disproportionately greater and more frequent consumption of sugar-sweetened beverages and foods (SSBF) would benefit from intervention strategies that are tailored to population-specific barriers and facilitators. The objective of this study was to develop and evaluate the acceptability of photo-enhanced and theory-based health promotion messages that target the reduction in SSBF among adult residents of public housing developments, a population known for their high rates of chronic disease. Using the message development tool as a framework, we developed a series of 15 SSBF reduction messages, using an iterative process with community member input. We then evaluated the acceptability of the messages and compared three delivery mechanisms: print, text, and social media. We recruited participants who were residents of urban public housing developments, and who spoke either English or Spanish. A majority of participants identified as being of Hispanic ethnicity (73%). The message acceptability scoring did not appear to differ according to the assigned delivery mechanism, despite some imbalances in participants’ characteristics across delivery mechanisms. The messages that targeted motivation were least likely to be accepted. In conclusion, our findings suggest that engaging members of the community at all phases of the development process was a feasible method to develop SSBF reduction messages with a high perceived acceptability.

## 1. Introduction

In the United States, it is estimated that 63% of people 18 years or older consume sugary drinks at least daily, thereby contributing to the development of chronic health conditions. At the national level, 63% of adolescents and 49% of adults drink at least one sugar-sweetened beverage on a given day [1]. Although sugar-sweetened beverages and sugary food (SSBF) consumption is a problem at a national level, low-income communities, as well as members of racial/ethnic minority groups, are more likely to consume sugar-sweetened beverages and sugary foods more frequently and in greater amounts, compared to those with higher incomes [2] and those not identifying as members of racial/ethnic minority groups. Given the disproportionate rates of consumption, intervention strategies aimed at reducing SSBF consumption for these communities would likely benefit from population-specific targeting of intervention strategies. 

The work reported on herein focuses on a population of adults residing in public housing in Boston, MA. Public housing is a nationwide governmental program that provides housing for low-income families [3]. Residents of public housing are predominantly members of racial/ethnic minority groups, and selection into housing is largely associated with a lower socioeconomic position, both of which have been associated with an increased risk of chronic disease [4]. Environmental factors, shaped by upstream factors such as structural racism, are associated with the physical locations of public housing developments, including lack of access to healthy foods, lack of green space/parks, and increased risks to physical safety which can further compound health status by posing additional barriers to healthy eating behaviors [5,6]. For these reasons, the chronic disease burden associated with residence in public housing developments is often higher than that of comparable populations outside of public housing [7]. Thus, the infrastructure of public housing offers an opportunity to develop tailored health promotion interventions that can benefit populations that have the highest burden of disease. 

An innovative method to promote healthy eating behaviors for communities at high risk of poor health outcomes is through the use of picture-based messages that target a reduction in risky behaviors at both the individual and community level. Picture-based messages have the potential to more effectively influence health behaviors of members of high-risk population groups through population-specific targeting of both health promotion messages and photos/pictures [8]. The message development tool is a social marketing approach that recognizes health messages as complex and dynamic, and provides a framework to develop health promotion messages using iterative cyclical feedback from the target audience [9]. Prior studies have used the message development tool framework to improve messages related to oral health [10] and diabetes care [11]. However, prior research has not applied this strategy to messages that aim to target healthy eating, specifically the reduction in SSBF consumption among residents of public housing. The aim of this study was to develop photo-enhanced and theory-based health promotion messages that target the reduction in SSBF among adults living in public housing. A second aim was to assess the acceptability of the developed messages and delivery preferences. 

## 2. Methods

**Figure 1:**
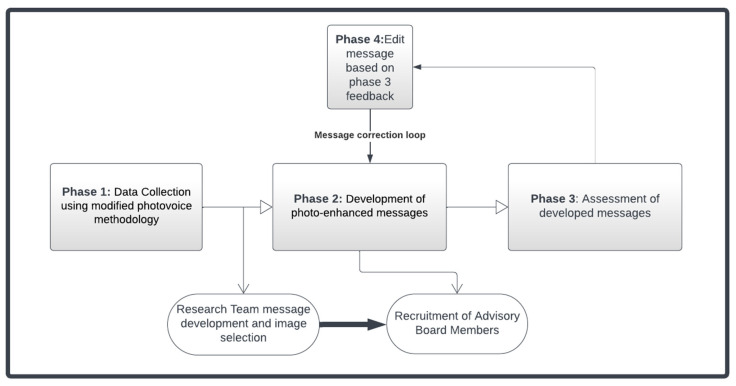
Multi-step process for health promotion message development using the message development tool framework.

This study followed a multi-phase approach according to the well-established message development tool [9] framework. Study participants included adult residents of two family-designated public housing developments in Boston, MA who spoke either English or Spanish. We discuss methods and results according to this multi-phased process (see Figure 1).

### 2.1. Phase 1: Data Collection Using Modified Photovoice Methodology

To understand barriers and facilitators faced by public housing residents in reducing SSBFs, we previously conducted a qualitative study using modified photovoice methodology (publication pending). Briefly, photovoice is a needs assessment method that enables members of a community to gather information about their environment through the use of photographs and narratives that explain what is happening in the photograph, as well as what it means to them and for future action [8]. We conducted a modified photovoice study among residents of Boston, MA public housing residents by training them in the use of photovoice methods and inviting them to apply the method to identify factors related to SSBF consumption through the following questions: “*What makes it hard to avoid sugary drinks and/or foods*? *What makes it easy to avoid sugary drinks and/or foods? Think about things in your life and in your community*”. Once participants collected images in response to the above questions, they were invited back to discuss how each of their photos related to their consumption of sugary foods and beverages. To analyze the content of the discussion, we used a qualitative, team-based thematic analysis approach [12]. We used an ecological framework depicting individual-, social-, physical-, and macro-level environmental influences on eating behaviors to organize our qualitative codes [13]. We then grouped similar codes together to create themes. We used NVivo version 12 as our qualitative data management platform. We found that, although the discussion questions posed to participants focused on sugary foods and beverages, participants often returned the discussion to factors that influenced healthy eating and cooking more generally. This leads us to posit that, in order to ultimately have an impact on sugary foods and beverage consumption, message design needs to focus on multi-level drivers of healthy eating, such as values, cultural traditions, and the home environments. Details on this study are pending publication. 

### 2.2. Phase 2: Development of Photo-Enhanced Messages

Using information gathered during phase 1 of the study, the research team developed 15 health promotion messages, with accompanying photos, that included advice and web links with helpful resources to decrease SSBF consumption. The content of these messages aimed to address the healthy eating themes identified in Phase 1. These messages were short, with a total of 140 characters and a photo accompanying each message. The character limit was guided by our desire to effectively deliver messages via both SMS text message and social media platforms such as Instagram©. Message creation was guided by the use of social cognitive theory [14], which encompasses interactions between people, their behaviors, and the environment, and is frequently used to underpin interventions targeting healthful eating and SSBF consumption [15,16,17]. Our messages targeted core social cognitive theory constructs of self-efficacy, motivation, and outcome expectations [14]. Of the 15 developed messages, a total of 5 messages targeted self-efficacy, 5 targeted motivation, and 5 targeted outcome expectations. Messages were initially developed in English and later translated into Spanish by two members of the research team who were native Spanish speakers. 

To review the content and effective tailoring of the developed messages, we convened a community advisory board. To construct this board, the Community Engagement Specialist member of our research team invited residents who were active in their communities and who spoke either English or Spanish. A total of seven residents joined our advisory board, three of whom considered themselves Hispanic and spoke Spanish. We asked advisory board members to participate in 5 focus groups. They were not required to come to all five meetings but were asked to attend at least three. Due to the COVID-19 pandemic, meetings happened virtually using Zoom from January to April 2021 and were recorded. Initially, members of the board rated each message according to their reactions to the content, relevance, usefulness, and impact on perceptions of self-efficacy, motivation, and outcome expectations using a scale of 1 through 7, where 1 was “not at all”, 4 “somewhat”, and 7 “very much”. Upon giving the rating, they were asked to explain why they gave the selected rating and to comment on the wording of the messages. We presented a series of pictures for each message and members of the board selected the photo that would accompany the message from two to three options. After reviewing English messages, we asked Spanish speaking participants to stay afterwards to rate and comment on the content of the Spanish version of the messages and to give a rating. At the end of this process, we had 15 completed photo-enhanced messages in English, and 15 of the same messages with the same photos available in Spanish. 

### 2.3. Phase 3: Assessment of Developed Messages

To evaluate the acceptability of the developed messages, we recruited 129 residents, from two, family-designated Boston public housing developments from June 2022 to October 2022, to receive each message via a preferred delivery channel over the course of approximately one month. Residents were eligible to participate if they spoke either English or Spanish and were 18 years of age or older. A stratified sampling approach was used to ensure that we sampled an approximately representative distribution of caregivers/non-caregivers of young children according to their relative distribution in the public housing population overall. Participant recruitment was primarily carried out via door-to-door knocking in which study staff members provided verbal and written explanations of the study to a responding adult and/or left an informational flyer that contained contact information for study staff members. Once we made initial contact, we provided verbal and written explanations of the study (e.g., informed consent). Study staff members documented verbal consent to participate in the study record. We approached every household across both participating developments to solicit participation. Recruitment attempts via door-to-door knocking and/or telephone contact for each household occurred up to three times during which the attempt time varied across weekdays/weekends and time of day. We documented recruitment outcomes for each household in the study record. This approach to recruitment (e.g., door-to-door knocking and informational flyers) has been associated with success in prior studies conducted within public housing populations [18].

Upon enrollment, participants were asked to complete a baseline survey. We collected demographic information, a brief screener of SSBF consumption and related attitudes, and message delivery preferences. Specifically, participants were asked to list their first and second preferred choice of delivery method. There were three modes of available delivery methods: printed messages (color-printed paper delivered to residence), SMS text messages to a personal phone, or social media-based messages (Facebook or Instagram). Each delivery method was selected based on their demonstrated promise as a means of intervention delivery in low-income populations [10,19]. Once enrolled in the study, each participant was randomized to either their first or second choice delivery preference and were notified of their selected delivery method. The photo-enhanced messages were identical across delivery channels. Individuals in the social media-based group were invited to be part of a private Facebook group or a private Instagram account. Messages on the private group were updated every 72 h, where we deleted old messages and added new ones. 

The order in which messages were presented to the participant was random to prevent any effects of ordering on message-specific ratings. Within 24 h of randomization to a delivery channel, participants received their first message and/or were added to a social media group where a message was available. Within 12–36 h of message delivery, we called participants to administer a post-message survey which included multiple questions, all using five-point Likert scales to globally assess participant understanding, acceptance, and satisfaction with the message text, as well as the overall appeal of the photos accompanying the message. Additionally, to measure each behavioral construct (e.g., self-efficacy, motivation, and outcome expectations) we asked the following questions (according to the message type): (1) how much did the message make you feel confident to eat food or drink beverages that are low in sugar? (2) How much did the message make you feel motivated to eat food or drink beverages that are low in sugar? (3) How much did the message make you think about the consequences of your food or beverage choices? Finally, after participants had received all 15 messages, they were asked to complete a short survey which asked about their overall satisfaction with the entire suite of 15 photo-enhanced messages. Participants were compensated for their time through provision of a $15 gift card after each completion of three post-message surveys, and a final overall acceptability survey (totaling $75). In addition, those who completed all 15 post-message surveys were entered into a raffle with 20 other completers for a $50 gift card. The methods and associated procedures for this message acceptability study, including a waiver of documentation of informed consent, were approved by the Boston University Medical Campus Institutional Review Board (protocol H-41656). 

### 2.4. Survey Measures 

Demographic information obtained on the baseline survey included race/ethnicity (Hispanic, Black non-Hispanic, White non-Hispanic, other), language spoken (English, Spanish or both), education (less than high school, high school graduate/GED, some college/technical school, college graduate) and employment status (full time, part time, or unemployed). Using brief measures of SSBF consumption commonly used for population surveillance, participants indicated SSBF consumption (rarely or never, weekly, once a day, twice a day, or three or more times a day) [17]. We also measured constructs of knowledge and self-efficacy for sugar-sweetened beverages consumption using a validated questionnaire for public housing residents [17]. We evaluated knowledge by creating an average of correct answers from four item questions. We also evaluated self-efficacy by taking the mean of the five-point scale across included items.

### 2.5. Data Analysis 

To evaluate the primary indicators of message acceptability, we generated descriptive statistics. We report means for continuous variables and frequencies for categorical variables. Outcome variables were stratified by delivery preference, delivery method, caregiver status, and by age, assessing any differences using a chi-square test. To identify whether any messages needed redevelopment or refinement, we created a summary score by taking the mean response across all intended outcomes, i.e., encouragement, satisfaction, relevance understandable, appeal, self-efficacy, motivation, and outcome expectation. The distribution of the mean ranking was evaluated and a redevelopment threshold determined. Any individual message that scored below the threshold qualified. 

### 2.6. Phase 4: Edit and Refine Messages

In accordance with the message development framework, we created a second advisory board, following completion of phase 3, in order to revisit the messages that scored below our selected threshold. Members of this board were also active leaders in their community, but did not participate as a board member in phase 2 of our study. A total of 5 members joined the meeting to review the selected messages. We asked participants to describe what they liked about the message, what they did not like, and what they would like to change in the message, including the accompanying photo. We took notes on the feedback and comments and discussed them as a team, updating messages and photos accordingly.

## 3. Results 

Table 1 displays the population characteristics of our sample, stratified by the assigned delivery mechanism. Participants were predominantly women, with approximately one-third identifying as caregivers of young children. A majority of participants identified as being of Hispanic ethnicity (73%), while 23% identified as Non-Hispanic Black (23%). In our sample, 28.4% indicated that they consumed sugary drinks at least once per day, with 15% reporting two or more per day. Sugary foods were reported less frequently by comparison, with 20.1% reporting at least daily consumption. A total of 56 people were randomized to receive messages via text message, 20 to social media, and 53 to paper, based on their top two preferences. A preference for message delivery via social media was the least common among participants, while a preference for paper-based messages was the most common. Participants were randomized to their first choice of delivery mechanism 55% of the time, and message scoring did not appear to differ according to assigned delivery mechanism or by stated preferences, despite some imbalances in participants’ characteristics across delivery mechanisms (see Table 1).

Average scores for each message across all measured domains (e.g., encouragement, satisfaction, etc.) ranged from 4.65 to 4.83 on a scale of 1 to 5 (see Table 2). In general, messages that aimed to target motivation had the lowest average scores overall. The average score for message relevance and the feelings of encouragement associated with the messages were lower compared to the feelings of satisfaction, understanding, and appeal. 

Stratified analyses based on delivery type and caregiver status revealed that average scores across all domains were lowest for those assigned to social media (4.71), followed by text messaging (4.76), and paper-based messages (4.81). Caregiver status appeared to influence message scoring. For example, among caregivers, messages involving children, such as making home-cooked meals with kids, were ranked most favorably. Whereas, among non-caregivers, the highest-scoring message pertained to the use of SNAP benefits to save money. Both groups, however, scored messaging about personal motivations to decrease dietary sugars as the lowest. 

In the post-satisfaction survey, the majority of participants indicated that the messages met all (32.4%) or some (64.9%) of their goals. Additionally, 94.6% indicated that the number of messages received were just right and 82.9% indicated that the messages were useful. Most participants indicated that, if messages were to become available to the community, 81.1% indicated that they are likely to share them with others. 

Table 3 displays the message topics, complete message text, and the photos used, as well as the theoretical construct the message targets. The message code was the shorter version of the messages which we referred to when conducting their post message survey. This table shows the final versions of the messages, including those that were revised as part of phase 4. 

## 4. Discussion

We developed a series of photo-enhanced, theory-based health promotion messages that targeted the reduction in sugar-sweetened beverages and foods among public housing residents. In all the phases of message development, we sought the active participation of community members, and applied the iterative process outlined in the message development tool framework, to guide message development. Overall, the participants received the messages with high acceptability. This may be a reflection of the incorporation of community member input throughout the message development process. These results are consistent with Bowen et al. [20] who suggest that, for public housing residents, the engagement of community leaders leads to the effective development of health promotion materials. Our finding that, overall, the messages were highly rated may also reflect the fact that we achieved a sufficient level of targeting of the important factors related to SSBF consumption as identified by residents of public housing in our target audience in phases 1 and 2. Several decades of research, as well as more recent systematic reviews [21,22], have demonstrated that individually tailored messages are more effective across a range of behaviors compared to general, non-tailored messages. For instance, caregivers of small children rated the messages involving children more favorably than non-caregivers, potentially reflecting the additional layer of tailoring based on caregiver status. However, this was not a universal finding, as we also found that the messages pertaining to SNAP use were more poorly rated, potentially reflecting that care should be taken before including SNAP use as a tailoring variable across public housing resident populations. 

Our findings demonstrated that messages targeting motivation were less likely to be perceived as highly acceptable. This may reflect a variation in levels of motivation among our sample. While motivation to decrease SSBF consumption was high overall at baseline, some research suggests that messages need to be specifically designed to reach those with low motivation, beginning with an understanding of factors leading to low motivational states in the first place [23]. Future work may wish to develop a range of motivational messages that target those with lower and higher motivation to change the target behavior. 

Although mean scores did not significantly vary by delivery method, our sample had a small number of participants who indicated social media as their first and second delivery choice. While we do not know for certain the reasons why most people did not choose social media as their preferred delivery method, data from the Pew Research Center show that Facebook and Instagram are two of the most used social media platforms among people with the demographic characteristics of our sample (e.g., women, those of Hispanic ethnicity, those reporting low-income [24]). Among residents of public housing specifically, research carried out by members of our research team also demonstrated that recent social media use is frequent [25]. Although social media use is frequent, use of online sources for health information may reflect a digital divide among population groups. For instance, data from the Health Information National Trends Survey indicate that income is a significant predictor of accessing health information online without frustration, with lower income indicating lower odds of accessing information easily [26]. We suggest that future research should explore the perceptions of barriers and facilitators to the use of social media platforms for health information among residents of public housing.

The limitations of our study should be considered. The majority of participants were women and of Hispanic ethnicity. Given our goal to create messages using participant preferences, our messages reflected their feedback and may not adequately represent the preferences of those from other demographic groups. Similarly, the high levels of acceptability may reflect a selection bias in that individuals agreeing to participate may already be health-conscious and in agreement with the themes reflected in the messaging. A strength of our study is the co-development of messages in English and in Spanish languages. The impact of the COVID-19 pandemic caused us to conduct phases 1, 2, and 4 via Zoom, which may have influenced how participants provided feedback compared to in-person groups. However, we feel robust feedback was provided by most participants who attended. In addition, attendance may have been facilitated by having groups held virtually.

Given that the majority of participants perceived that the suite of messages were useful and they would be willing to share them with others, we believe a future potential avenue for message dissemination is through social networks. Intervention research conducted in public housing settings supports the feasibility and preliminary efficacy of approaches that recognize the importance of environmental factors, including the social environment [19]. Social network interventions may be particularly well suited to public housing settings given the physical structure of the buildings and the existing social relationships among residents. Our findings suggest that multiple delivery mechanisms should be considered, from print, text, or social media, and that an iterative planned message development process is a promising way to create SSBF consumption messages that resonate with residents of urban public housing.

## Figures and Tables

**Table 1:** Population characteristics stratified by delivery type.

		Delivery Method		
	Total (N = 129)	Text (N = 56)	Social Media (N = 20)	Paper (N = 53)
**Characteristics**				
Female (n(%))	120 (93.0)	53 (94.6)	19 (95.0)	48 (90.6)
Age (n(means))	126 (45.9)	54 (46.03)	19 (42.53)	52 (47.01))
Caregiver (n(%))				
*Yes*	41 (32.0)	18 (32.1)	9(47.4)	14 (26.4)
*No*	87 (68.0)	38 (67.9)	10 (52.6)	39 (73.6)
Language (n(%))				
*English*	37 (29.1)	19 (33.9)	7 (36.8)	11 (21.2)
*Spanish*	61 (48.0)	26 (46.4)	7 (36.8)	28 (53.9)
*Both English and Spanish*	29 (22.8)	11 (19.6)	5 (26.3)	13 (25.0)
Race/Ethnicity (n(%))				
*Black non-Hispanic*	30 (23.4)	15 (26.8)	5 (25.0)	10 (19.2)
*White non-Hispanic*	4 (3.1)	0 (0)	1 (5.0)	3 (5.8)
*Hispanic*	93 (72.7)	40 (71.4)	14 (70.0)	39 (75.0)
*Other*	1 (0.8)	1 (2)	0 (0)	0 (0.8)
USA (n(%))	63 (50.8)	28 (50.0)	13 (68.4)	24 (45.3)
Education (n(%))				
*Less than High School*	17 (13)	8 (14.3)	1 (5.0)	8 (15.1)
*High school graduate/GED*	73 (57)	31 (55.4)	10 (50.0)	32 (60.4)
*Some College/technical School*	33 (25)	15(26.8)	6(30.0)	12 (22.6
*Some college*	17 (13)	7 (12.5)	5 (25.0)	5 (9.4)
*College Degree*	6 (5)	2 (3.6)	3 (15.0)	1 (1.9)
Employment Status (n(%))				
*Full Time*	24 (19)	12 (21.4)	3 (15.0)	9 (17.0)
*Part time*	23 (18)	10 (17.9)	5 (25.0)	8 (15.1)
*Unemployed*	82 (64)	34 (60.7)	12 (60.0)	36 (67.9)
Frequency of sugary drinks (n(%))				
*Rarely or never*	58 (44.9)	28 (50.0)	6 (30.0)	24 (45.3)
*At least once a week but not every day*	34 (26.4)	13 (23.2)	7 (35.0)	14 (26.4)
*Once a day*	17 (13.2)	9 (16.1)	2 (10.0)	6 (11.3)
*Two or more times a day*	20 (15.5)	6(10.7)	5 (25.0)	9 (17.0)
Frequency of Sugary Foods (n(%))				
*Rarely or never*	53 (41.1)	23 (41.1)	9 (45.0)	21 (39.6)
*At least once a week but not every day*	50 (38.8)	24 (42.9)	9 (45.0)	17 (32.1)
*Once a day*	14 (10.9)	4 (7.1)	1 (5.0)	9 (17.0)
*Twio or more times a day*	12 (9.2)	5 (8.9)	1 (5.0)	6 (10.8)
Knowledge Questions				
Recommended sugary drinks (n(%))	97 (75)	42 (75.0)	16 (80.0)	39 (73.6)
Natural sugar, such as honey, is healthier for teeth than white sugar (n(%))	19 (15)	6 (10.9)	4 (20.0)	9 (17.0)
The sugar that is found naturally in fruits and milk is the same as the sugar that is added to foods and drinks when they are being prepared (n(%))	17 (13)	8 (14.3)	2 (10.0)	7 (13.2)
Sugar that is found in 100% fruit juice is healthier than the sugar that is added to make soda (n(%))	28 (22)	15 (26.8)	5 (25.0)	8 (15.1)
Knowledge about sugary beverages, score (mean (sd))	1.24 (0.75)	1.25 (0.70)	1.35 (0.88)	1.19 (0.76)
Self-efficacy on Sugary Drinks (mean (sd))	3.84 (1.31)	3.77 (1.34)	3.75 (1.37)	3.9 (1.26)
Self-efficacy on Sugary Foods (mean (sd))	3.82 (1.31)	3.64 (1.36)	4.24 (1.14)	3.85 (1.28)
Smoking Status (n(%))				
*Daily smoker*	17 (13)	6 (10.7)	5 (25.0)	6 (11.3)
*Non-daily smoker*	5 (4)	2 (3.6)	1 (5.0)	2 (3.8)
*Former smoker*	8 (6)	4 (7.1)	0	4 (7.6)
*Never smoked*	99 (77)	44 (78.6)	14 (70.0)	41 (77.4)
Self-rated dental health status (n(%))				
*Excellent/Very Good*	11 (8.52)	6 (10.71)	2 (10.00)	3(5.66)
*Good*	74 (57.36)	34 (60.71)	12 (60.00)	28 (52.83)
*Fair/Poor*	42 (32.55)	16 (28.58)	5 (25.00)	21 (39.62)
*Subject is edentulous*	2 (1.55)	0	1 (5.00)	1 (1.89)
Dental Cleaning (n(%))				
*Within the past 12 months*	112 (87)	50 (89.3)	18 (90.0)	44 (83.0)
Self-rated health status (n(%))				
*Excellent/Very Good*	22 (17.1)	9 (16.1)	3(15.0)	10 (18.8)
*Good*	72 (55.8)	33 (58.9)	13 (65.0)	26 (49.1)
*Fair/Poor*	35 (27.2)	14 (25.00)	4 (20.0)	17 (32.1)
Summary Score for Message Targets (mean (sd))				
*Motivation*	4.72 (0.72)	4.59 (0.85)	4.60 (0.77)	4.85 (0.53)
*Outcome Expectations*	4.82 (0.55)	4.80 (0.58)	4.72 (0.63)	4.68 (0.51)
*Self-Efficacy*	4.80 (0.59)	4.76 (0.63)	4.74 (0.56)	4.86 (0.56)
First Choice Preference	71 (55.47)	55 (98.21)	3 (15.00)	13 (25.00)
Second Choice Preference	57 (44. 53)	1 (1.79)	17 (85.00)	39 (75.00)

**Table 2:** Averages Scores of Messages.

	Encouraged	Satisfaction	Relevance	Understanding	Appeal	Photos Rating	Self-Efficacy	Motivation	Outcome-Expectations	Means
Health benefits of decreasing sugar	4.82	4.93	4.71	4.97	4.86	4.87	.	.	4.84	4.86
Goals to improve health conditions	4.7	4.86	4.89	4.95	4.83	4.88	4.84	.	.	4.85
Teaching kids healthy recipes	4.75	4.92	4.75	4.91	4.81	4.87	.	.	4.83	4.84
Packing food to save time	4.74	4.89	4.73	4.94	4.84	4.9	4.77	.	.	4.83
Making home cook meals with kids	4.71	4.89	4.69	4.93	4.87	4.92	.	4.77	.	4.83
Using SNAP to save money	4.65	4.86	4.73	4.93	4.84	4.91	4.81	.	.	4.82
Making smoothies	4.71	4.93	4.6	4.95	4.83	4.86	4.81	.	.	4.81
Eating smaller dessert portion with your family	4.65	4.89	4.71	4.92	4.76	4.81	4.82	.	.	4.79
Making traditional meals to promote health	4.65	4.87	4.66	4.92	4.73	4.78	.	.	4.8	4.77
Grabbing water to decrease daily sugar	4.7	4.83	4.75	4.85	4.7	4.75	.	.	4.84	4.76
Cooking large meals to eat through the week *	4.6	4.73	4.59	4.98	4.72	4.87	.	4.77	.	4.75
Passing down recipes *	4.54	4.77	4.65	4.9	4.78	4.87	.	4.69	.	4.74
Cost of buying coffee everyday *	4.64	4.78	4.61	4.92	4.69	4.81	.	4.68	.	4.73
The amount of sugar in your soda *	4.78	4.75	4.63	4.88	4.61	4.75	.	.	4.79	4.73
Personal motivations to decrease sugar *	4.53	4.79	4.67	4.75	4.53	4.61	.	4.66	.	4.65

* Messages have been modified to reflect suggestions made in phase 4.

**Table 3:** Final message text in English with the final selected photos.

Construct	Short Code	English	Selected Photo
Motivation	Cost of buying coffee everyday *	Sugary coffee or tea may seem like an inexpensive treat that helps keep you going during the day, but in the long run, sugary drinks cost you more in terms of health conditions, medications & wellbeing! Try coffee or tea with less sugar	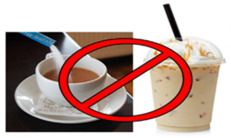
Motivation	Cooking large meals to eat through the week *	There are lots of ways to save time AND eat healthy meals low in sugar. Instead of take-out fast food, do home-cooked fast food! Cook a large meal to eat throughout the week or divide it into smaller meals and freeze to have later during the week.	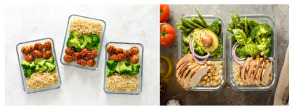
Motivation	Making home cook meals with kids	Making home cooked meals colorful with fruits & veggies is fun! Kids may be more likely to eat healthy low sugar meals you make together. Make it art or a game! See what kinds of faces, animals, or shapes you can make. Snap and share a picture of your favorite creation.	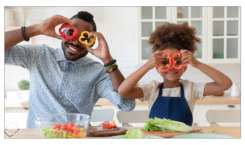
Motivation	Passing down recipes *	Focus on meals low in sugar that keep people healthy. Look for recipe inspiration here: https://bit.ly/3t0LXNi (accessed on 15 May 2023).Try a healthy low-sugar breakfast; for example, a burrito, oatmeal with fruit or mangu (mashed plantains) with eggs & veggies instead of pancakes & syrup.	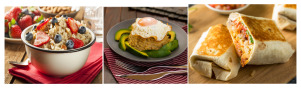
Motivation	Personal motivations to decrease sugar *	Think about what motivates you to eat less sugar & lower your risk of future health conditions: Being a good role model? Enjoying time with your family? Being more self confident? Whatever your motivations are—use them to make healthy changes!	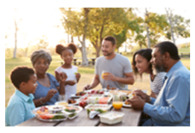
Outcome Expectation	The amount of sugar in your soda *	Did you know? Buying one bottle of soda OR one coffee per day adds up to about 480 spoonfuls of sugar and $68 per month! Try buying packs of water or sugar-free beverages like seltzer, flavored water, tea or diet beverages to save your money and your teeth :-D	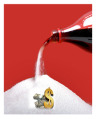
Outcome Expectation	Grabbing water to decrease daily sugar	Feeling thirsty when you’re out and about? Try grabbing no sugar options like water, seltzer & unsweetened tea or coffee. Cutting out 1 bottle of regular soda reduces your daily sugar intake by 16 spoonfuls! Less sugar is good for your teeth, heart, and weight!	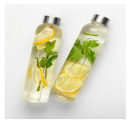
Outcome Expectation	Making traditional meals to promote health	Traditional meals bring cultural values to your table. Swap your favorite healthy recipes with family and friends! To promote heart health, use less butter when you cook. Keep things flavorful by adding garlic and spices like pepper.	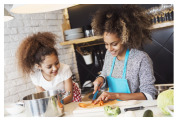
Outcome Expectation	Teaching kids healthy recipes	Teaching kids to make healthy family recipes can help them avoid health conditions like diabetes later in life. For quick & easy recipes to try with your family check out: https://foodhero.org/kids (accessed on 15 May 2023).	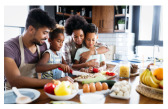
Outcome Expectation	Health benefits of decreasing sugar	There are lots of benefits you can expect by eating foods & drinks low in sugar. Benefits include: improved weight and healthier teeth; lowered risk of heart disease and diabetes. That’s a lot of good for your body!	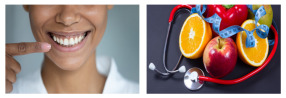
Self-Efficacy	Using SNAP to save money	There are lots of ways to make fruits & veggies less expensive: try farmer’s markets, sales, coupons, or buying frozen and canned options. If you receive SNAP, you can use your benefits to buy fruits & veggies and get money back on your card. Learn more: https://bit.ly/3iQessv (accessed on 15 May 2023)	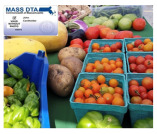
Self-Efficacy	Packing food to save time	Eating healthy snacks doesn’t have to take a lot of time. Try packing a piece of fruit and a small handful of nuts in to-go containers. Now your snack is ready to grab when you’re on your way out. It’s quick, healthy & filling!	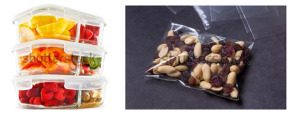
Self-Efficacy	Making smoothies	Love smoothies? Make them yourself at home to control the ingredients & the sugar. Follow 3 tips: (1) Start with whole fruits & veggies (2) Avoid juice (use milk or water), (3) Add protein & creaminess (try plain Greek yogurt, oats, peanut butter, or soft tofu)	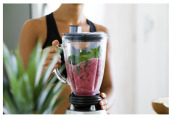
Self-Efficacy	Eating smaller dessert portion with your family	Start a healthier dessert tradition with your family! Serve a smaller portion of dessert, fill the rest of the plate with cut up fruit.	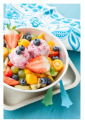
Self-Efficacy	Goals to improve health conditions	To improve or lower your risk of health conditions like diabetes, try setting healthy eating goals each week. Post your goals on your fridge to remind yourself! For example, replace a sugary dessert with fruit or have seltzer instead of soda	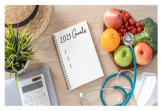

* Message and/or photo was revised during Phase 4 of the message development process.

## Data Availability

The data used in this study are available upon reasonable request to the corresponding author.
